# The Food Contaminants Nivalenol and Deoxynivalenol Induce Inflammation in Intestinal Epithelial Cells by Regulating Reactive Oxygen Species Release

**DOI:** 10.3390/nu9121343

**Published:** 2017-12-11

**Authors:** Simona Adesso, Giuseppina Autore, Andrea Quaroni, Ada Popolo, Lorella Severino, Stefania Marzocco

**Affiliations:** 1 Department of Pharmacy, University of Salerno-Via Giovanni Paolo II, 132-84084 Fisciano-Salerno, Italy; sadesso@unisa.it (S.A.); autore@unisa.it (G.A.); apopolo@unisa.it (A.P.); 2 Department of Biomedical Sciences, Cornell University, Veterinary Research Tower, Ithaca, NY 14853-6401, USA; aq10@cornell.edu; 3 Department of Veterinary Medicine and Animal Production, Division of Pharmacology and Toxicology, University of Naples Federico II, via Delpino 1, 80137 Naples, Italy; lseverino@unina.it

**Keywords:** nivalenol, deoxynivalenol, intestinal epithelial cells, inflammation, oxidative stress, mycotoxin combination

## Abstract

*Fusarium* mycotoxins are fungal metabolites whose ability to affect cereal grains as multi-contaminants is progressively increasing. The trichothecene mycotoxins nivalenol (NIV) and deoxynivalenol (DON) are often found in almost all agricultural commodities worldwide. They are able to affect animal and human health, including at the intestinal level. In this study, NIV, both alone and in combination with DON, induced inflammation and increased the inflammatory response induced by lipopolysaccharide (LPS) plus Interferon-γ (IFN) in the non-tumorigenic intestinal epithelial cell line (IEC-6). The inflammatory response induced by NIV and DON involves tumor necrosis factor-α (TNF-α) production, inducible nitric oxide synthase (iNOS) and cyclooxygenase-2 (COX-2) expression, nitrotyrosine formation, reactive oxygen species (ROS) release, Nuclear Factor-κB (NF-κB), Nuclear factor (erythroid-derived 2)-like 2 (Nrf2) and inflammasome activation. The pro-inflammatory effect was strongly induced by NIV and by the mycotoxin mixture, when compared to DON alone. Mechanistic studies indicate a pivotal role for ROS in the observed pro-inflammatory effects induced by mycotoxins. In this study, the interactions between NIV and DON point out the importance of their food co-contamination, further highlighting the risk assessment process that is of growing concern.

## 1. Introduction

Type B trichothecenes are a group of mycotoxins that mainly contaminate cereals following fungal infection by *Fusarium* species, namely *F. graminearum* and *F. culmorum* [1]. The simultaneous exposure to several thricotecene mycotoxins often occurs, in humans as well as in animals. The co-contamination is due to the ability of most *Fusarium* to simultaneously produce more mycotoxins. Moreover, food and feed can be contaminated by various fungi at the same time, or in quick succession. A diet generally consists of different food and feed components from cereals, thus also contributing to the simultaneous mycotoxins exposure [2]. In a study of *Fusarium* in food samples in the European Union, 57% of samples were positive for deoxynivalenol (DON) and 16% were positive for nivalenol (NIV) [3]. DON is especially seen as an important food safety issue since it is the most prevalent mycotoxin in Europe and North America [3,4,5]. A high number of consumers have been reported to be exposed to DON at levels close to, or even higher than, the daily intake limit [5,6]. Although consumers do not consider NIV to be a risk since the daily intake remains below the tolerable daily intake level [7], evidence suggests both the stronger toxic effect of NIV, in addition to DON, and the implications of their combined effects, may be of concern [8,9,10,11]. Due to their presence in the food chain these mycotoxins are potentially hazardous for human health, affecting various organs and systems, such as the gastrointestinal tract. Experimental studies reported that low to moderate acute oral exposure to trichothecenes causes vomiting, diarrhoea, and gastroenteritis, whereas higher doses cause severe damage to the lymphoid and epithelial cells of the gastrointestinal mucosa, resulting in hemorrhage, endotoxemia and shock. Chronic exposure to trichothecenes can cause anorexia, reduced weight gain, diminished nutritional efficiency, neuroendocrine changes, and immune modulation [12,13]. 

After ingestion, the first host defence barrier against mycotoxins is the intestinal epithelium. To protect the intestine and the entire body from inflammatory and infectious disease, the healthy functioning of the epithelial barrier and innate immunity are important. Intestinal epithelial cells (IECs) are then exposed to a variety of external stresses, including food-derived stimulants, and generating a proper response to these stimulants is one of the major roles of the epithelial cells. To respond to these external stimulants, IECs and immune cells are cooperatively activated, thereby producing cytokines and other bioactive compounds that reinforce and restore the intestinal barrier. These protective responses may, however, simultaneously induce inflammation [14]. During the active disease phase, the production of pro-inflammatory cytokines and chemokines, and the induction of oxidative reactions by activated leukocytes and epithelial cells, are the main events occurring in intestinal inflammation. Reactive oxygen species (ROS) and their oxidized by-products regulate redox-sensitive signaling pathways and transcription factors, which sustain inflammation within the intestinal layer. Thus, the intestinal epithelium is an inflammatory tissue by nature, always maintaining a moderate inflammatory state. This type of inflammation in normal intestines is mild and controllable, and is, therefore, called “controlled inflammation”. However, if inflammatory reactions immoderately proceed because of excessive stress or the formation of a vicious reaction cycle, disruption of the epithelial tissues and dysfunction of the intestines will occur. A typical and severe example of such uncontrollable inflammation is inflammatory bowel disease (IBD), which includes Crohn’s disease and ulcerative colitis [15]. The precise etiology of IBD remains unclear, although it is likely multifactorial involving a number of elements leading to the generation of chronic inflammation and development of IBD, including Crohn’s disease (CD) and ulcerative colitis (UC). A hypothesis would be that at least in some cases, the ingestion of food contaminated with mycotoxins could be also involved in inducing inflammatory bowel diseases [16]. IECs are especially sensitive to NIV and DON and exposure to these toxins may induce toxicity as apoptosis, oxidative stress and impaired barrier function [10,11,17,18]. Moreover, it has been hypothesized that human exposure to DON may play an important role in the etiology of various chronic IBD [19].

Previous studies reported the ability of DON to induce inflammation, evaluating some inflammatory mediators at the mRNA level or on tumorigenic cell lines. Less is known about NIV or its effects, as co-contamination, when combined with DON on inflammatory response in IECs [20,21,22]. Thus, the aim of our study was to evaluate the NIV and DON ability, alone and in combination, to induce and/or exacerbate an inflammatory response in the non-tumorigenic intestinal epithelial cells (IEC-6).

## 2. Materials and Methods

### 2.1. Reagents

Unless stated otherwise, all reagents and compounds were purchased from Sigma Chemicals Company (Sigma, Milan, Italy). 

### 2.2. Cell Culture

The IEC-6 cell line (CRL-1592) was purchased from the American Type Culture Collection (ATCC, Rockville, MD, USA). The IEC-6 cells originated from normal rat intestinal crypt cells [23]. Cells were cultured using Dulbecco’s modified Eagle’s medium (DMEM, 4 g/L glucose) supplemented with 10% (*v*/*v*) heat-inactivated foetal bovine serum (FBS), 2 mM l-glutamine, 1.5 g/L NaHCO_3_, and 0.1 unit/mL bovine insulin. Cells were used between the 17th and 21st passages for the experiments.

### 2.3. Cell Treatment

The IEC-6 cells were plated and, after 24 h, were treated with NIV and DON, either alone or in combination, at varying concentrations (0.5–5 μM), for different times, as outlined below. In another set of experiments, the IEC-6 cells were incubated with NIV and DON, alone or in combination (0.5–5 μM) for 1 h and then exposed simultaneously to mycotoxins and lipopolysaccharides from *E. coli* (LPS; 10 μg/mL) and Interferon-γ (IFN; 10 U/mL) for different times, as outlined below. In order to evaluate the possible contribution of NIV and DON-induced ROS on some inflammatory mediators in some experiments, diphenyleneiodonium chloride (DPI; 10 μM), an antioxidant, was added 1 h before NIV and DON cell treatment.

### 2.4. Tumor Necrosis Factor and Interleukin 1β Determination

The TNF-α and IL-1β levels in the IEC-6 cellswere assessed with an Enzyme-Linked Immuno Sorbent Assay (ELISA. IEC-6 cells were plated into 24-well plates (8.0 × 10^4^ cells/well) and allowed to adhere for 24 h. Cells were then treated with the mycotoxins, as previously indicated, for 24 h. The cellular supernatants were then collected and diluted 1:5 with the assay diluent 1× (e-Biocscience, San Diego, CA, USA) and the ELISA assays were performed on these cellular supernatant by using a commercial kit, according to the manufacturer’s instructions (e-Biosciences, San Diego, CA, USA). Results were expressed as pg/mL as previously reported [24].

### 2.5. Measurement of Cyclooxygenase 2, Inducible Nitric Oxide Synthase, Heme Oxygenase 1, and Caspase-1 by Cytofluorimetry

For these evaluations, IEC-6 cells were plated into 96-well plates (1 × 10^4^ cells/well), allowed to adhere, and treated as indicated for 24 h. The IEC-6 cells were then collected, washed with phosphate buffer saline (PBS), then incubated in Fixing Solution for 20 min and then in Fix Perm Solution for 30 min. Anti-COX-2 (BD Transduction Laboratories, Milan, Italy), anti-iNOS (BD Transduction Laboratories), anti-HO-1 (Santa Cruz Biotechnologies, Dallas, TX, USA), or anti-caspase-1 (Abcam, Cambridge, UK) antibodies were then added. The cells were then treated with the secondary antibody, in Fix solution, and cell fluorescence was evaluated using a fluorescence-activated cell sorter (FACSscan; Becton Dickinson, Milan, Italy) and analyzed with Cell Quest software (Becton Dickinson, Milan, Italy) as previously reported [25].

### 2.6. Measurement of Intracellular ROS Release

ROS levels were evaluated by means of the probe 2′,7′-dichlorofluorescin-diacetate (H_2_DCF-DA) [26]. The IEC-6 cells were plated in 24-well plates (8 × 10^4^ cells/well). After adhesion, cells were treated with NIV and DON, alone, in a mixture, or in the presence of LPS + IFN, for 24 h. The IEC-6 cells were then collected, washed with PBS buffer, and then incubated in PBS containing H_2_DCF-DA (10 μM). After 15 min at 37 °C, cell fluorescence was evaluated using a fluorescence-activated cell sorter (FACSscan; Becton Dickinson, Franklin Lakes, NJ, USA) and analyzed with Cell Quest software (Becton Dickinson, Milan, Italy).

### 2.7. Immunofluorescence Analysis for Nitrotyrosine, Nuclear Factor-Like 2, and Nuclear Factor-kB with Confocal Microscopy

IEC-6 cells (2 × 10^5^ cells/well) were seeded on coverslips in a 12-well plate and treated with NIV and DON (5 μM) for 1 h, alone or in a mixture, or in the presence of LPS + IFN for 24 h for nitrotyrosine formation and for 1 h for Nuclear Factor-kB (NF-κB ) and Nuclear Factor (erythroid-derived 2)-like 2 (Nrf2) activation. In some experiments, DPI (10 μM) was added 1 h before NIV and DON incubation. After, the treatment cells were fixed with 4% paraformaldehyde in PBS and permeabilized with 0.1% saponin in PBS. After blocking with bovine serum albumin (BSA) and PBS, cells were incubated with rabbit anti-nitrotyrosine (Millipore, Billerica, MA, USA), rabbit anti-Nrf2 antibody (Santa Cruz Biotechnologies, Dallas, TX, USA), or rabbit anti-phospho p65 NF-κB (Santa Cruz Biotechnologies, Dallas, TX, USA), for 1 h at 37 °C. The slides were then washed three times with PBS and fluorescein-conjugated secondary antibody (FITC) was added for 1 h. 4′,6-diamidine-2′-phenylindole dihydrochloride (DAPI) was used for the counterstaining of nuclei. Lastly, coverslips were mounted in mounting medium and fluorescent images were taken under the Laser Confocal Microscope (Leica TCS SP5, Leica, Wetzalar, Germany) as previously reported [27].

### 2.8. Data Analysis

Data are reported as mean ± standard error mean (s.e.m.) values of at least three independent experiments, each completed in triplicate. Statistical analysis was performed by analysis of variance test, and multiple comparisons were made by Bonferroni’s test. A *p*-value less than 0.05 was considered significant.

## 3. Results

### 3.1. NIV and DON Increased TNF-α Production in IEC-6 Cells

**Figure 1:**
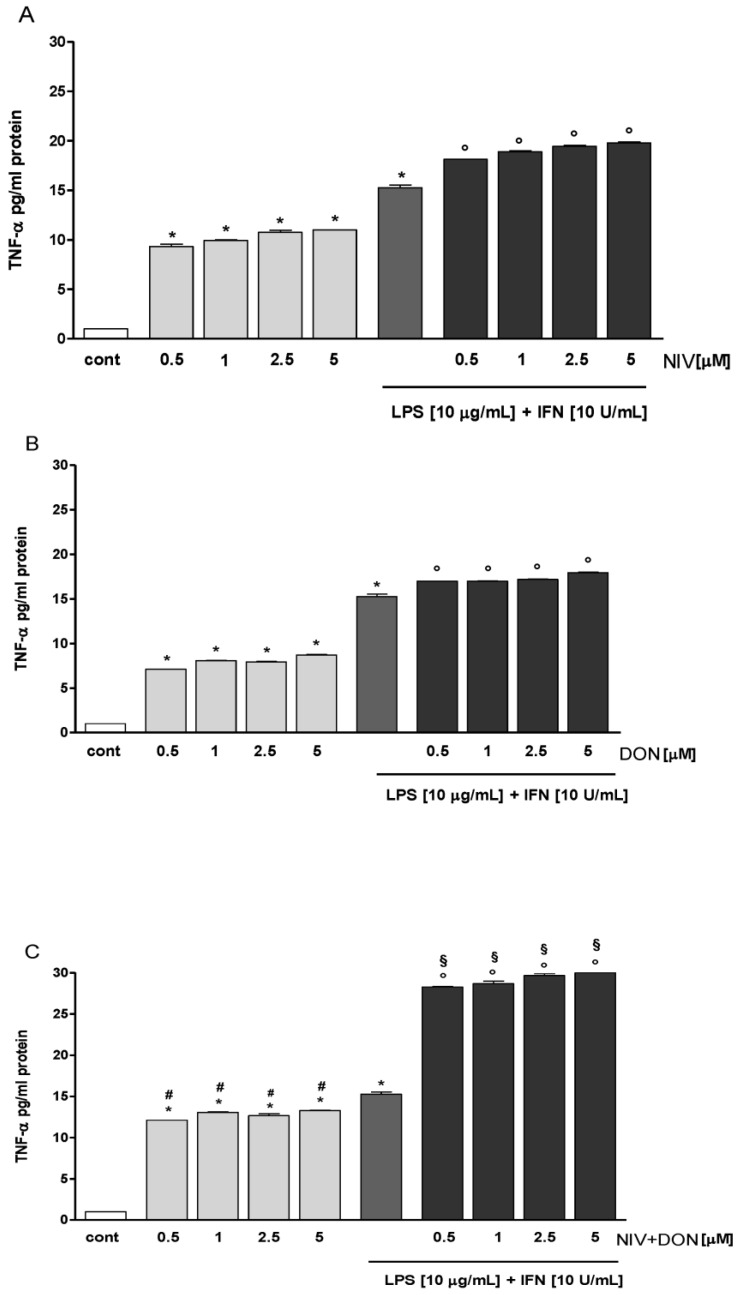
(**A**) Effect of nivalenol (NIV); (**B**) deoxynivalenol (DON); and (**C**) their combination (NIV + DON) in normal and in inflammatory conditions on tumor necrosis factor-α (TNFα) levels in the IEC-6 cellular medium, evaluated by Enzyme-Linked Immuno Sorbent Assay (ELISA) assay. Values are expressed as pg/mL protein or mean fluorescence intensity. * Denotes *p* < 0.05 vs. control; ^°^ denotes *p* < 0.05 vs. LPS + Interferon-γ (IFN); ^#^ denotes *p* < 0.05 vs. DON alone; and ^§^ denotes *p* < 0.05 vs. DON + LPS + IFN.

To investigate the effect of NIV, DON, and NIV + DON (0.5–5 μM), alone, or in combination with LPS and IFN, on TNF-α levels in IEC-6 cellular medium, we performed an ELISA assay. Our results showed that NIV, DON, and their mixture significantly increased TNF-α levels at all tested concentrations, with a *p*-value < 0.05 vs. the control and a *p*-value < 0.05 vs. DON alone (Figure 1A–C). In particular, during inflammatory conditions in the IEC-6 cells, we observed a further increase in mycotoxin-induced TNF-α production at all tested concentrations with *p* < 0.05 vs. the control (Figure 1A–C).

Moreover, NIV significantly increased DON-induced TNF-α release during inflammation (*p* < 0.05 vs. DON, LPS, and IFN) (Figure 1C).

### 3.2. NIV and DON Induced COX-2 and iNOS Expression in IEC-6 Cells 

**Figure 2:**
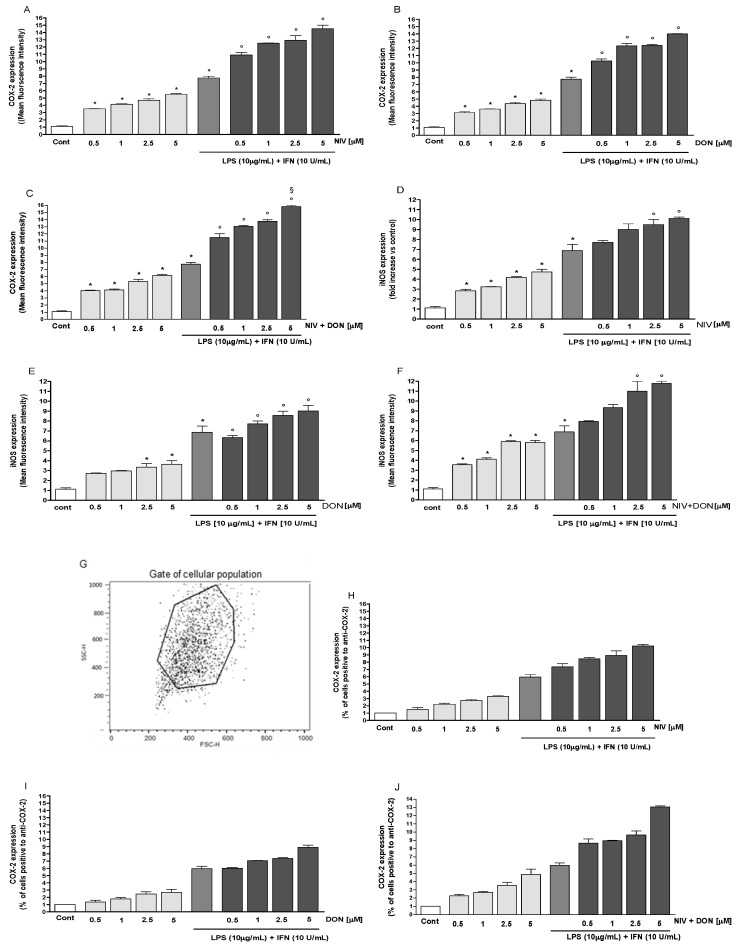

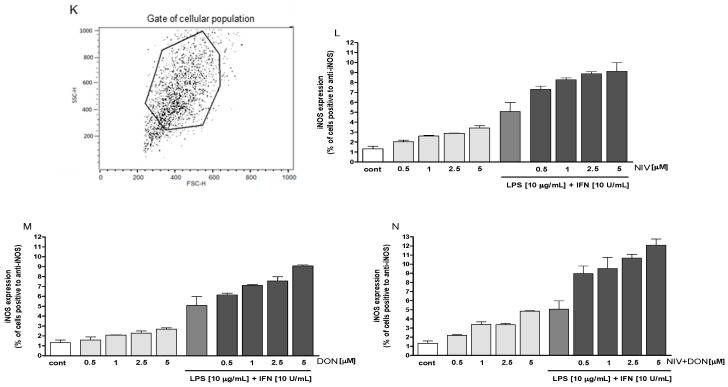
(**A**) Effect of NIV; (**B**) DON; and (**C**) their combination (NIV + DON) in normal and in inflammatory conditions on cyclooxygenase-2 (COX-2) expression, evaluated by cytofluorimetric technique. Values are expressed as mean fluorescence intensity; (**D**) Effect of NIV; (**E**) DON; and (**F**) their combination (NIV + DON) on inducible nitric oxide synthase (iNOS) expression, evaluated by cytofluorimetric technique. * Denotes *p* < 0.05 vs. control; ^°^ denotes *p* < 0.05 vs. lipopolysaccharide (LPS) + Interferon-γ (IFN), respectively; ^§^ denotes *p* < 0.05 vs. DON + LPS + IFN; (**G**) Flow cytometry figures showed gated cells for COX-2 (**H**–**J**) Histograms representing the percentage of cells positive to anti-COX-2; (**K**) Flow cytometry figures showed gated cells for iNOS; (**L**–**N**) Histograms representing the percentage of cells positive to anti-iNOS.

Our evaluation of pro-inflammatory factors indicated that cyclooxygenase-2 (COX-2) expression increased with both the addition NIV (*p* < 0.05 vs. control) (Figure 2A) and DON (*p* < 0.05 vs. control) (Figure 2B). This effect was enhanced when the mycotoxin mixture was added to the IEC-6 cells (*p* < 0.05 vs. control) (Figure 2C). When the mycotoxins, either alone or in combination, were added with LPS and IFN to the IEC-6 cells, we observed a further increase in COX-2 expression (*p* < 0.05 vs. LPS and IFN; *p* < 0.05 vs. DON, LPS, and IFN) (Figure 2A–C). NIV, DON, and NIV and DON also increased inducible nitric oxide synthase (iNOS) expression mostly during the inflammatory condition, and especially when added in combination to the IEC-6 cells (*p* < 0.05 vs. control; *p* < 0.05 vs. LPS and IFN) (Figure 2D–F). 

### 3.3. NIV and DON Increased Nitrotyrosine Formation and ROS Release in LPS and IFN γ Treated IEC-6 Cells

**Figure 3:**
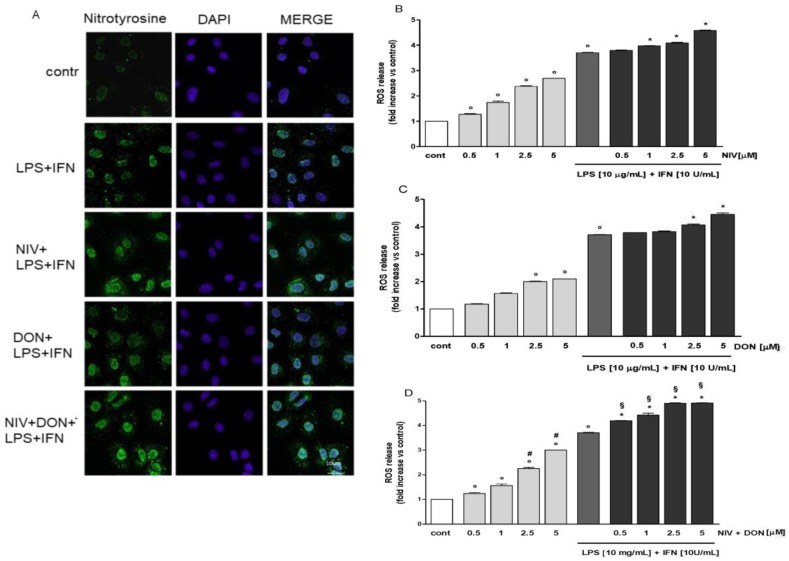
(**A**) Effects of NIV, DON, and their combination (NIV + DON; 5 μM), in inflammatory conditions on nitrotyrosine formation were evaluated using immunofluorescence assay confocal microscopy. Scale bar: 10 μm. Blue and green fluorescences indicate the location of the nucleus 4′,6-diamidine-2′-phenylindole dihydrochloride (DAPI) and nitrotyrosine, respectively; Effect of (**B**) NIV; (**C**) DON; and (**D**) their combination (NIV + DON) on ROS formation, evaluated with the probe 2′,7′ dichlorofluorescein-diacetate (H_2_DCF-DA). Values, mean ± s.e.m., are expressed as mean fluorescence intensity. ^°^ Denotes *p* < 0.05 vs. control. * Denotes *p* < 0.05 vs. LPS + IFN. ^#^ Denotes *p* < 0.05 vs. DON alone. ^§^ Denotes *p* < 0.05 vs. DON + LPS + IFN.

iNOS expression, leading to nitric oxide production, induces peroxynitrite formation, and 3-nitrotyrosine formation is the hallmark product. NIV and DON (5 μM) induced an increase of nitrotyrosine formation in the IEC-6 cells. This effect was greater when the IEC-6 cells were treated with both mycotoxins together (Figure 3A). To investigate the effect of the mycotoxins under inflammatory conditions and on the oxidative stress in the IEC-6 cells, the intracellular ROS production was evaluated. NIV and DON induced a significant release of ROS in IEC-6. In the presence of LPS and IFN, a significant increase in ROS production at the two highest mycotoxin concentrations (2.5 and 5 μM) was observed (*p* < 0.05 vs. LPS and IFN) (Figure 3B,C). Interestingly, when NIV and DON were simultaneously added to the IEC-6 cells, they induced a significant increase in ROS release at all tested concentrations, and especially in inflammatory conditions (*p* < 0.05 vs. control; *p* < 0.05 vs. LPS and IFN; *p* < 0.05 vs. DON alone; and *p* < 0.05 vs. DON, LPS, and IFN) (Figure 3D).

### 3.4. NIV and DON Induce Nrf2 Activation HO-1 Expression in IEC-6 Cells

**Figure 4:**
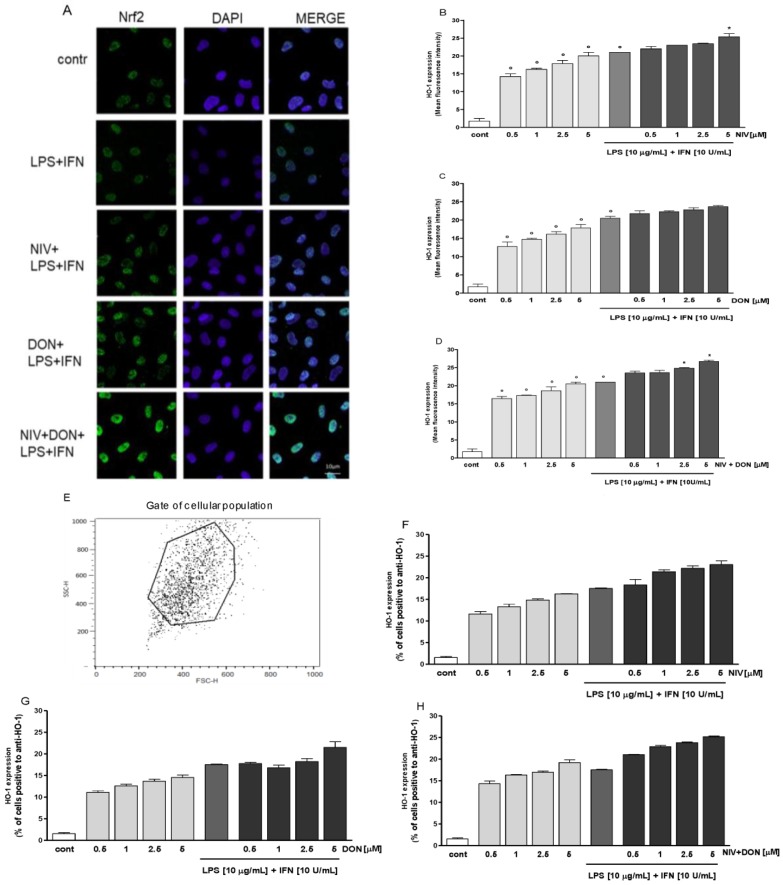
(**A**) Effect of NIV, DON, and their combination (NIV + DON; 5 μM), on Nrf2 nuclear translocation, evaluated using immunofluorescence assay confocal microscopy. Scale bar: 10 μm. Blue and green fluorescences indicate localization of nucleus (DAPI) and Nrf2, respectively; (**B**) Effect of NIV; (**C**) DON; and (**D**) their combination (NIV + DON) on HO-1 expression in the IEC-6 cells, evaluated by cytofluorimetric technique. Values, mean ± s.e.m., are expressed as mean fluorescence intensity. ^°^ Denotes *p* < 0.05 vs control. * Denotes *p* < 0.05 vs. LPS + IFN; (**E**) Flow cytometry figures show gated cells for HO-1; (**F**–**H**) Histograms representing the percentage of cells positive to anti-HO-1.

To track the influence of NIV and DON on Nrf2 activation, we labelled Nrf2 with a green fluorescent probe. As shown in Figure 4A, nuclear Nrf2 was increased after one hour with the addition of NIV and DON. In addition, Nrf2 increased more so due to NIV when compared to DON, and increased the most with the addition of both mycotoxins to the IEC-6 cells. 

The expression of the HO-1 cytoprotective enzyme significantly increased in normal conditions, but during inflammation, a significant increase was observed for NIV only at the highest tested concentration (*p* < 0.05 vs. LPS and IFN) (Figure 4B) and for the higher tested concentrations for the combination of NIV and DON (*p* < 0.05 vs. LPS and IFN) (Figure 3D).

### 3.5. NIV and DON Induced p65 NF-κB Nuclear Translocation in IEC-6 Cells

**Figure 5:**
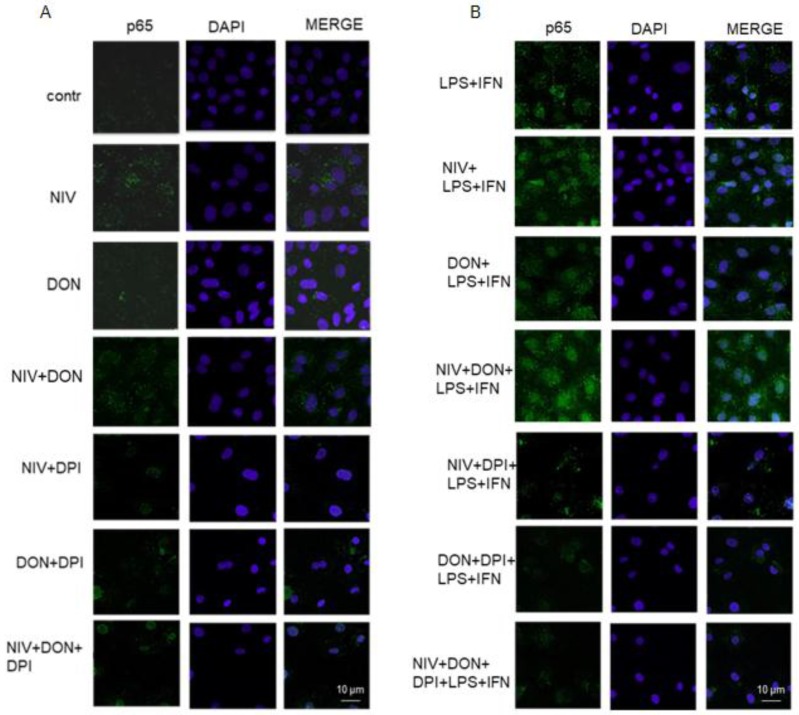
Effect of NIV, DON, and their combination (NIV + DIV; 5 μM) in normal (**A**) and in inflammatory conditions (**B**), also in presence of DPI, on NF-κB p65 nuclear translocation, evaluated using immunofluorescence assay confocal microscopy. Scale bar: 10 μm. Blue and green fluorescences indicate localization of the nucleus (DAPI) and NF-κB p65, respectively.

NF-κB p65 was labelled with green fluorescence to track the influence of the mycotoxins, at a concentration of 5 μM, added one hour before and simultaneously with LPS and IFN, to the IEC-6 cells, on its nuclear translocation. As shown in Figure 5, NIV and DON both increased NF-κB nuclear translocation under inflammatory conditions compared to LPS and IFN alone. Under the same experimental conditions, we examined NF-κB nuclear translocation in the presence of DPI (10 μM), added to the IEC-6 cells one hour prior to the mycotoxins. When DPI was added to the IEC-6 cells, in the presence of NIV, DON, and NIV and DON, under both normal and in inflammatory conditions, we observed a decrease of p65 NF-κB nuclear translocation (Figure 5).

### 3.6. NIV and DON Induced Inflammasome Activation in IEC-6 Cells

**Figure 6:**
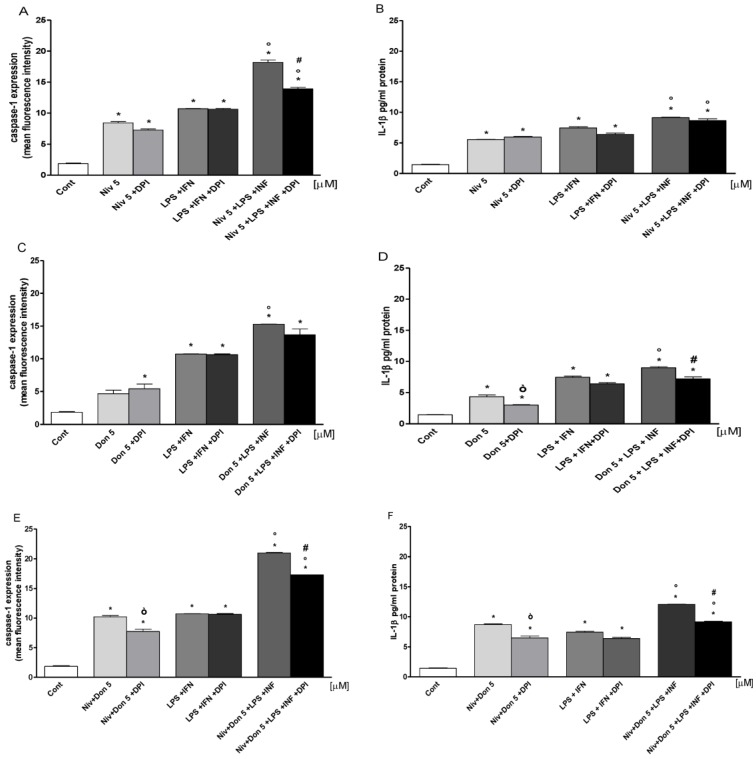
(**A**) Effect of NIV; (**C**) DON; and (**E**) their combination (NIV + DON; 5 μM) under normal and in inflammatory conditions on caspase-1 expression in IEC-6 cells, evaluated by cytofluorimetric technique; (**B**) Effect of NIV; (**D**) DON; and (**F**) their combination (NIV + DON; 5 μM) under normal and in inflammatory conditions on IL-1β levels in the IEC-6 cellular medium, evaluated by ELISA assay. Values are expressed as mean fluorescence intensity and as pg/mL protein. * Denotes *p* < 0.05 vs. control. ^°^ Denotes *p* < 0.05 vs. LPS + IFN. ò Denotes *p* < 0.05 vs. mycotoxin alone. ^#^ Denote *p* < 0.05 vs. mycotoxin + LPS + IFN.

NIV and DON, both alone and combined, induced inflammasome activation by increasing caspase-1 expression and IL-1β production in the IEC-6 cells (Figure 6). We observed that NIV (Figure 6A) and DON (Figure 6C), added one hour prior and simultaneously with LPS and IFN for 24 h, increased caspase-1 expression in IEC-6, under both normal and inflammatory conditions (*p* < 0.05 vs. control and *p* < 0.05 vs. LPS and IFN). The combination of mycotoxins (Figure 6E) resulted in a further increase in caspase-1 expression in the IEC-6 cells (*p* < 0.05 vs. control, *p* < 0.05 vs. LPS and IFN) (Figure 6E). In the same experimental conditions, DPI (10 μM), resulted in a decrease in caspase-1 expression, mostly in the IEC-6 cells treated with the mixture of NIV and DON (*p* < 0.05 vs. mycotoxin alone and *p* < 0.001 vs. mycotoxin, LPS, and IFN) (Figure 6A,C,E).

Similarly, NIV, DON, and their combination, significantly increased IL-1β levels, and in the presence of DPI, a reduction in IL-1β was observed (*p* < 0.05 vs. control; *p* < 0.05 vs. mycotoxin alone; *p* < 0.05 vs. LPS and IFN; and *p* < 0.05 vs. mycotoxin, LPS, and IFN) (Figure 6B,D,F).

## 4. Discussion

The mycotoxins’ ability to contaminate food and feed could influence both human and animal health. The main finding of this study is the pro-inflammatory effect of NIV and DON, both alone and in combination, and their ability to exacerbate the inflammatory response in IEC-6 cells. These results highlight the role of NIV and DON, mostly as food co-contaminats in intestinal inflammation and suggested the pivotal role of ROS in inducing the observed mycotoxins-induced pro-inflammatory effects in IECs. This study provides evidence that NIV and DON influence, both under normal and in inflammatory conditions: (i) TNF-α production; (ii) COX-2; (iii) iNOS; and (iv) HO-1 expression; (iv) nitrotyrosine formation; (v) ROS release; (vi) Nrf-2; (vii), NF-κB; and (viii) inflammasome activation. 

As a defense mechanism, IECs produce several pro-inflammatory cytokines, such as TNF-α. Recent studies strongly suggest that TNF-α is one of the major pathogenic cytokines involved in the pathogenesis of IBD as elevated levels of TNF are present in the serum of IBD patients [28]. In addition, an elevated number of TNF-secreting cells in the inflamed mucosa of IBD patients has been repeatedly reported [29,30,31]. Herein, *lamina propria* mononuclear cells isolated from colonic biopsies from IBD patients spontaneously produced increased amounts of TNF which correlated with the degree of tissue involvement and mucosal inflammation, strengthening the importance of TNF in the inflamed gut [32]. In our experimental model, NIV and DON, separately but even more so when combined, induce TNF-α release and further increase its production during LPS and IFN-induced inflammation in IEC-6. These data align with previous studies reporting the effect of DON in inducing TNF-α release in various experimental models [33,34]. Prior to this study, only one study was completed on TNF-α mRNA levels, while examining the NIV effects on TNF-α [35]. 

Together with TNF-α, pro-inflammatory inducible enzymes, such as COX-2 and iNOS, are predominantly expressed at the sites of inflammation, which may affect colon integrity and contribute to the development of intestinal damage [36]. Our data show that COX-2 protein expression significantly increased with the addition NIV and DON, alone and when combined, at all tested concentrations, both under normal conditions and strongly in the presence of pro-inflammatory stimuli. COX-2 expression was also influenced by iNOS; the main NOS isoform was expressed during inflammation and an interaction between iNOS and the COX pathway represents an important mechanism for the modulation of the inflammatory response [37]. Moreover, under inflammatory conditions, the expressed iNOS releases nitric oxide (NO) that rapidly reacts with superoxide anions, generating the toxic metabolite peroxiynitrite. Peroxynitrite is able to nitrate tyrosine residues in proteins, resulting in the formation of nitrotyrosine. Because the nitration of tyrosine is an alternative to phosphorylation at key residues, it can affect a protein’s enzymatic activity and interfere with intracellular signalling processes [38]. 

iNOS upregulation and nitrotyrosine immunoreactivity have been demonstrated in conditions such as endotoxemia, IBD, *Helicobacter pylori* gastritis, as well as in animal models of colitis and ileitis, suggesting that peroxynitrite may be a key mediator of mucosal injury and gut barrier failure [39]. We previously reported that NIV induces iNOS expression and nitrotyrosine formation, more than DON does, and that it increases DON-induced iNOS expression [11]. Here we report the stronger NIV and mycotoxin-combination effect, compared to DON alone, in inducing iNOS expression and nitrotyrosine formation under inflammatory conditions in IEC-6 cells. The gastrointestinal tract is a key source of ROS. Despite the protective barrier provided by the epithelial layer, ingested materials and pathogens can cause inflammation by activating the epithelium, polymorphonuclear neutrophils, and macrophages, to produce inflammatory cytokines and other mediators that further contribute to oxidative stress [40]. 

Various gastrointestinal pathological conditions, including gastroduodenal ulcers, malignancies, and irritable bowel disease, arise in part from oxidative stress. Both NIV and DON induce ROS release. Under inflammatory conditions, both mycotoxins exert a significant pro-oxidant effect only at the highest tested concentration, while when combined, they significantly increase ROS release at all tested concentrations, highlighting their pro-oxidant effect mainly during inflammation. A recurrent theme in oxidant signalling and antioxidant defence is reactive cysteine thiol-based redox signalling. Nrf2 is a regulator of cellular resistance to oxidants. Nrf2 controls the basal and induced expression of an array of element-dependent antioxidant response genes that regulate the physiological and physiopathological outcomes of oxidant exposure [41,42]. During inflammation, the mycotoxins, mostly when combined, enhance Nrf-2 activation, and thus an oxidative stress response, in IEC-6 cells. Despite this, under normal conditions, NIV and DON are able to induce significant HO-1 expression, a cytoprotective enzyme produced by Nrf-2 activation, in accordance with previous studies [11]. Here, we report that during inflammation, cells showed a significant increase in this cytoprotective factor only at the higher tested concentrations, leading to an oxidative stress condition.

NF-κB is primarily known as a potentially pathogenic factor that is harmful to the host when excessively or improperly activated. Moreover, an increase in ROS levels could promote NF-κB activation and its nuclear translocation [43]. This ROS/NF-κB self-sustaining regulatory loop may contribute to the progression of an uncontrolled inflammatory response [44]. It also performs a critical role in colorectal cancer initiation [45]. The ability of NIV and DON to influence NF-κB activation has been previously reported [11,46] and in this study, we report that the mycotoxins, individually and more so in combination, further increase NF-κB activation during inflammation. This effect seems to be strongly related to the mycotoxins’ ability to induce ROS release. DPI, a NADPH inhibitor, treatment in the IEC-6 cells reduced the mycotoxin-induced NF-κB activation, both under normal and inflammatory conditions, indicating the importance of the contribution of ROS in NF-κB activation in IEC-6 cells. 

The NLRP3 (NLR family, pyrin domain-containing 3) inflammasome regulates the interaction with other innate immune processes in the intestinal mucosa. The NLRP3 inflammasome is a multiprotein, cytoplasmic complex, composed of a NLR protein, the adaptor ASC, and procaspase-1, which regulates processing and secretion of cytokines belonging to the IL-1 family. The NLRP3 protein is widely expressed in the gastrointestinal tract and can be found in epithelial cells at mucosal sites [47]. The NLRP3 inflammasome may be stimulated by the presence of microbial-associated products, toxins, and by a vast range of endogenous and exogenous danger-associated signals, serving as a general sensor of any form of cellular stress triggered by signalling intermediates, including ROS [48]. Inflammasome facilitates the autocatalytic cleavage of pro-caspase-1 into its active form [49]. Active caspase-1 mediates acute inflammation by activating some inflammatory cytokines, such as IL-1β and IL-18 and it also can induce an inflammatory form of cell death, known as pyroptosis, which limits intracellular pathogen replication [50,51]. It has been reported that IL-1β and IL-18 levels are elevated in serum of IBD patients, furthermore, the levels of these cytokines correlated with the severity of intestinal inflammation, suggesting potential involvement of these cytokines in disease [52,53].

Although TLR signalling may be required for transcriptional up-regulation of the IL-1β precursor in some cell types, such as macrophages, NLRP3-mediated caspase-1 activation by TLR agonists (e.g., LPS or heat-killed bacteria) can proceed through pannexin-1, independent of the classical TLR pathway [54]. Signalling pathways via TLRs and distinct NLRs may converge in NF-κB activation, regulating the expression of numerous immune and inflammatory genes [55]. 

In our experimental study, NIV and DON increased IL-1β production and caspase-1 expression both in basal and inflammatory conditions, indicating the inflammasome activation in IEC-6 cells induced by these mycotoxins, mainly when combined. As the common feature of NLRP3 inflammasome activators is their ability to induce ROS production [56], we hypothesise that NIV and DON can induce inflammasome activation through ROS release, as indicated by the use of DPI in our experiments. Given the importance of IL-1β in mediating inflammation, deregulated inflammasome activation is implicated in the pathogenesis of a variety of inflammatory diseases [54].

A compromised barrier function is associated with increased epithelial permeability and translocation of luminal allergens and pathogens, as well as with a non-specific inflammatory response and an overstimulation of the gut-associated immune system [17]. In a previous study, we reported that both NIV and DON affected IEC migration (restitution), representing the initial step in gastrointestinal wound healing in the gut [10]. Thus, NIV and DON could induce intestinal inflammation either directly, through the stimulation of the production of inflammatory factors by the intestinal epithelium, as reported here, or indirectly, through alterations of the intestinal barrier function, allowing the transepithelial passage of luminal antigens. The pro-inflammatory effect, also with particular attention to inflammasome activation, has been recognized as the cause of chronic intestinal and non-intestinal disease [57]. Moreover, NIV and DON pro-inflammatory effects were also able to potentiate the action of other proinflammatory signals, such as LPS, significantly aggravating the existing inflammatory state, such as, for example, IBD. 

## 5. Conclusions

Our results indicate that in IECs: (i) NIV and DON significantly induce an inflammatory status and increase the pro-inflammatory response, mainly regulating ROS release and that (ii) NIV exerts a stronger effect respect to DON in these cells. Moreover, considering the importance of assessing DON toxicity also in the context of mycotoxin mixture [16], our data provide evidence that the DON toxicity is further enhanced in NIV co-contaminated food. These observations are of significance from the perspective that food contaminated with NIV and DON is a risk factor for intestinal inflammatory conditions, such as IBD. In addition, the range of concentrations used in this study for NIV and DON are consistent with the levels plausibly encountered in the gastrointestinal tract after consumption of heavily contaminated food [58].
